# The Effect and Safety of Xuefu Zhuoyue Prescription for Coronary Heart Disease: An Overview of Systematic Reviews and Meta-Analyses

**DOI:** 10.1155/2022/9096940

**Published:** 2022-11-17

**Authors:** Hongshuo Shi, Zunhao Tang, Ting Liu, Xuecheng Zhang, Yao Wang, Jie Li, Chengda Dong, Wenqiang Chen, Ruirui Hou, Guomin Si, Yan Liu

**Affiliations:** ^1^College of Traditional Chinese Medicine, Shandong University of Traditional Chinese Medicine, Jinan, China; ^2^Department of Traditional Chinese Medicine, Shandong Provincial Hospital Affiliated to Shandong First Medical University, Jinan, China; ^3^Key Laboratory of Chinese Internal Medicine of Ministry of Education, Dongzhimen Hospital, Beijing University of Chinese Medicine, Beijing, China; ^4^Beijing University of Chinese Medicine, Beijing, China; ^5^First Clinical School of Medicine, Shandong University of Traditional Chinese Medicine, Jinan, China; ^6^The Key Laboratory of Cardiovascular Remodeling and Function Research, Chinese Ministry of Education and Chinese Ministry of Health, The State and Shandong Province Joint Key Laboratory of Translational Cardiovascular Medicine, Cheeloo College of Medicine, Shandong University, Qilu Hospital of Shandong University, Jinan, China

## Abstract

**Background:**

In China, the traditional Chinese medicine compound Xuefu Zhuoyue prescription (XFZY) has been widely used in the therapy of coronary heart disease (CHD). Currently, several systematic reviews (SRs)/meta-analyses (MAs) of XFZY for the treatment of CHD have been published. This overview aims to evaluate the existing SRs/MAs and provide a scientific basis for evaluating the efficacy and safety of XFZY for the therapy of CHD.

**Methods:**

The SRs/MAs of XFZY for the treatment of CHD were obtained from 7 electronic databases with the search date set at March 7, 2022. Two researchers independently assessed the methodological quality, reporting quality, and evidence quality of the included SRs/MAs using the following tools: the Assessment of Multiple Systematic Reviews 2 (AMSTAR-2), the Preferred Reporting Items for Systematic Reviews and Meta-Analyses 2020 (PRISMA 2020), and the Grading of Recommendations Assessment, Development, and Evaluation (GRADE) system.

**Results:**

A total of 11 SRs/MAs were included in this overview. All SRs/MAs assessed by means of AMSTAR-2 had more than one critical defect, so all SRs/MAs were rated low. Regarding the assessment of reporting quality, the results of PRISMA 2020 showed that none of the SRs/MAs were fully reported. In addition, the results of the GRADE assessment of the quality of evidence indicated that only one outcome was rated as high quality across all SRs/MAs.

**Conclusion:**

Current evidence suggests that XFZY is effective and safe for the management of patients with CHD. However, the high risk of bias of the original clinical studies and the low quality of the SRs/MAs reduced the reliability of the results.

## 1. Introduction

Coronary heart disease (CHD), one of the most common cardiovascular diseases, is mainly caused by obesity, diabetes, and smoking [1], and it has become a major risk factor for death and disability worldwide [2]. CHD is characterized by the formation of arterial plaques mainly composed of lipids, inflammatory cells, and calcium [3], and these plaques cause the constriction or spasm of the coronary lumen, eventually leading to myocardial ischemia, hypoxia, and even necrosis [4, 5]. In addition to age and gender, risk factors for coronary heart disease include abnormal lipid metabolism, hypertension, hyperlipidemia, and obesity [6]. Although the use of antiplatelet agents and statins has significantly reduced the incidence of adverse cardiovascular events, drug dependence and residue as well as the long-term risk of coronary heart disease remain unresolved issues [7]. Therefore, it is urgent to search for a more effective treatment [8].

With unique advantages and significant clinical efficacy [9], traditional Chinese medicine (TCM) has been used for thousands of years in the treatment of CHD and related diseases. Xuefu Zhuoyue prescription (XFZY) was originally founded by Qingren Wang, a famous doctor in the Qing Dynasty, and it has been a formula commonly used in TCM for the treatment of cardiovascular diseases [10]since then. XFZY consists of 11 kinds of herbs including *Achyranthes bidentata* Bl (“Niuxi” in Chinese, NX), *Ligusticum chuanxiong* Hort (“Chuanxiong” in Chinese, CX), *Paeonia lactiflora* Pall (“Chiao” in Chinese, CS), *Angelica sinensis* (Oliv.) Diels (“Danggui” in Chinese, DG), *Glycyrrhiza inflata* Bat (“Gancao” in Chinese), *Carthamus tinctorius* L (“Honghua” in Chinese, HH), *Bupleurum chinense* DC (“Chaohu” in Chinese, CH), *Prunus persica* (L.). Batsch (“Torn” in Chinese, TR), *Platycodon grandiflorus* (Jacq.) A. DC (“Jiegeng” in Chinese, JG), *Citrus aurantium* L. (“Zhiqiao” in Chinese, ZQ), and *Rehmannia glutinosa* Libosch (“Dihuang” in Chinese, DH). Several small trials have found that XFZY was safe and effective in CHD treatment, manifested in improving angina symptoms and myocardial ischemia with fewer side effects [11, 12]. Animal studies have shown that XFZY can reduce intracellular adhesion molecule-1 (ICAM-1) and vascular cell adhesion molecule-1 (VCAM-1), thereby reducing the inflammatory response induced by ischemia-reperfusion injury (IRI) [13].

Over the past 10 years, there have been a number of systematic reviews (SRs)/meta-analyses (MAs) that focused on assessing the potential benefits of XFZY for the health management of patients with CHD. However, the methods and quality of the evidence for their work have not been assessed, which may mislead clinicians in actual decision-making [14]. The overview is a new research methodology for assessing the quality of multiple SRs/MAs in an effort to resolve the inconsistencies in the evidence and identify key gaps in the use of the evidence [15]. We, therefore, conducted this study to evaluate the evidence of XFZY for CHD treatment in the real-world implementation arena. We assessed methodological quality, reporting quality, and evidence quality of relevant SRs/MAs by the Assessment of Multiple Systematic Reviews 2 (AMSTAR-2), the Preferred Reporting Items for Systematic Reviews and Meta-Analyses 2020 (PRISMA 2020), and the Grading of Recommendations Assessment, Development, and Evaluation (GRADE) system.

## 2. Methods

This research was conducted according to the Cochrane Handbook and some high quality articles with scientific research methodologies [16–18]. This overview protocol has been registered with the INPLASY website (Registration number: INPLASY202260077).

### 2.1. Eligibility Criteria

Eligible studies meet the following criteria: (1) type of research: SRs/MAs of randomized controlled trials (RCTs) reported the efficacy or safety of XFZY in CHD treatment; (2) inclusion of the population: patients diagnosed as having CHD based on diagnostic criteria regardless of age, nationality, or gender; (3) interventions: the control group intervention was conventional treatment (CT) with no other herbal medicines. According to the guidelines, CT should be routine medicines that inhibit angina pectoris, thrombosis, platelet aggregation, arrhythmias, hypertension, and diabetes as well as statins. The intervention method for the experimental group was XFZY or XFZY combined with the medicines received by the control group; (4) outcomes: clinical efficiency rate, relief of anginal symptoms (RAS), electrocardiogram (ECG), left ventricular end-systolic diameter (LVESD), left ventricular ejection fraction (LVEF), endothelin-1 (ET-1), nitric oxide (NO), ICAM-1, C-reactive protein (CRP), VCAM-1, superoxide dismutase (SOD), malondialdehyde (MDA), creatine kinase-MB (CK-MB), brain natriuretic peptide (BNP), angina frequency (AF), plasma viscosity (PV), whole blood viscosity (WBV), duration of angina pectoris (DAP), fibrinogen (FB), high-density lipoprotein cholesterol (HDL-C), total cholesterol (TC), triglyceride (TG), low-density lipoprotein cholesterol (LDL-C), and adverse event (AE). Clinical efficiency rate, RAS, and ECG are defined in Supplementary File 1.

Studies that met the following criteria were excluded: (1) network meta-analyses, SRs/MAs without meta-analysis, review articles, conference abstracts, editorials, case reports, and replication studies; (2) animal experiments; (3) the control group using any other traditional Chinese medical method.

### 2.2. Search Strategy

Two researchers (HS–S and ZH-T) independently searched PubMed, Embase, Cochrane Library, CBM, CNKI, Wanfang database, and VIP database on March 7, 2022. A search strategy featuring the combination of keywords and free words was adopted, where the keywords include “Xuefu Zhuoyue,” “coronary heart disease,” “meta-analysis,” and “systematic review.” The search strategy was adjusted to fit the different databases. In addition, we manually searched for relevant references to ensure the completeness of the search. The search strategy for PubMed was shown in Table 1, and search strategies for other databases are shown in Supplementary File 2.

### 2.3. Literature Screening

Two independent researchers (WQ-C and RR-H) conducted the screening of the literature. The retrieved publications were imported into a literature management system (EndNote X9), and the initial screening was performed by firstly removing the duplicates and subsequently reading the titles and abstracts. Finally, the full-text was read to identify the final literature for inclusion.

### 2.4. Data Extraction

To ensure data integrity and consistency, the two researchers (ZH-T and HS-S) used a predesigned data extraction table to extract the data. The extracts included the following: first author and year of publication (country), number of RCTs (number of subjects), interventions, risk of bias assessment methods, interventions, and main findings.

#### 2.4.1. Quality Evaluation for Inclusion in SRs/MAs

Two independent researchers (HS–S and CD-D) assessed the methodological quality, report quality, and evidence quality of the included SRs/MAs. Any disagreements were referred to a third investigator (Y-L) for consultation.

#### 2.4.2. Methodological Quality Evaluation

The methodological quality of the included SRs/MAs was assessed using the AMSTAR-2 [19]. The tool contains seven key items (2, 4, 7, 9, 11, 13, and 15). Each item was categorized as “no,” “partially yes,” or “yes” depending on their adherence to the criteria. The overall methodological quality was classified into four levels: high, medium, low, or extremely low.

#### 2.4.3. Report Quality Evaluation

The PRISMA 2020 [20] was used to assess the quality of the report and it covers 27 items. Each item can be assessed as “yes,” “partially yes,” or “no,” with a ratio based on the assessment of each item.

#### 2.4.4. Evidence Quality Evaluation

The GRADE [21] system was applied to assess the quality of evidence for inclusion in the SRs/MAs outcome indicators. Evidence quality may be downgraded due to the following 5 criteria: risk of bias, inconsistency, indirectness, imprecision, and publication bias. The quality of evidence was categorized as high, moderate, low, and extremely low. The evidence with less than one degradation factor is rated as high quality, while the evidence with one degradation factor is rated as medium quality, two degradation factors are rated as low quality, and more than three (including three) degradation factors are rated as extremely low quality.

### 2.5. Data Synthesis

Narrative descriptions were given for the included SRs/MAs. Dichotomous variables are expressed as risk ratios (RR) or odds ratios (OR) with 95% confidence intervals (CI), while continuous variables are expressed as standardized mean differences (SMD) or mean differences (MD) with 95% CI. In addition, the results of the AMSTAR 2, PRISMA 2020, and GRADE assessments are shown in the table.

## 3. Results

### 3.1. Literature Selection

**Figure 1:**
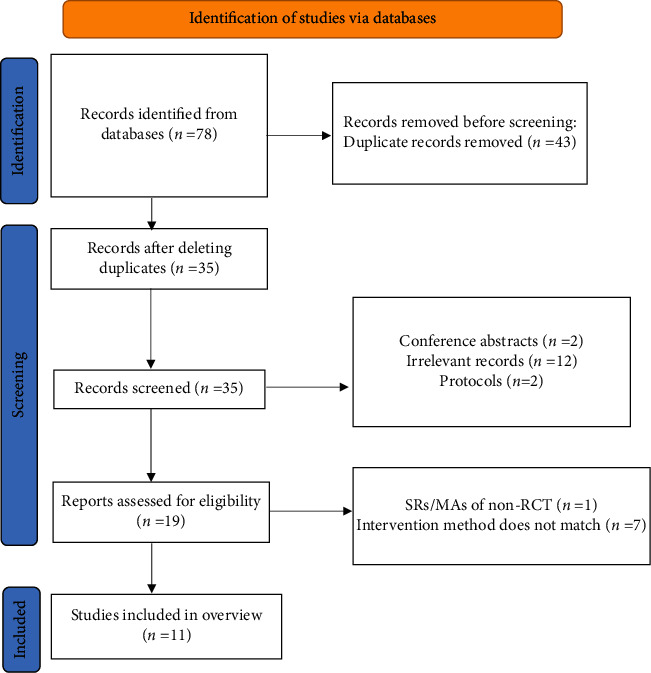
The flowchart of the screening process.

A total of 78 publications were obtained from seven electronic databases after the search; among those, 45 were excluded after duplicates removal, 17 were excluded by screening the titles and abstracts, and 7 [22–28] were further excluded after the full text was read due to their failure to meet the intervention criteria. Finally, 11 publications [29–39] were included for the study. The flow chart of literature screening is shown in Figure 1.

### 3.2. Characteristics of the SRs/MAs

The characteristics of the 11 SRs/MAs used for qualitative analysis in this overview were summarized in Table 2. All SRs/MAs were published between 2014 and 2022, with 6 (6/11, 54.5%) [30, 31, 34, 35, 37, 38] of them being published within the last 5 years. All the included SRs/MAs were published by Chinese scholars, five [29–33] of which were in English and six [34–39] in Chinese. The number of RCTs included per SR/MA ranged from 8 to 30, and the participants in these RCTs ranged from 534 to 3,126. In terms of intervention modality, CT was used in the control group, while XFZY was used in the experimental group or added to the control group. Seven SRs/MAs [29–34] used the Cochrane criteria for risk of bias assessment of included RCTs, and the remaining 4 SRs/MAs [35, 36, 38, 39] used the Jadad scale. All SRs/MAs were subjected to meta-analysis and all reported positive results.

### 3.3. Quality Assessment

#### 3.3.1. Methodological Quality Assessment

AMSTAR-2 was used to assess the methodological quality of the SRs/MAs included in this research, the details of which are given in Table 3. Due to multiple deficiencies in critical and noncritical items, the methodological quality of all SRs/MAs was low. The deficiencies in the inclusion of SRs/MAs assessed by AMSTAR-2 were as follows: Item 2 (only 2 SRs/MAs [26, 27] have registered study protocols), Item 7 (none of the SRs/MAs provided a list of excluded articles), and Item 10 (none of the SRs/MAs provided a list of funding for RCTs).

### 3.4. Report Quality Assessment

Detailed information on the quality of the report was presented in Table 4. Although the titles, abstracts, introductions, and discussions of the SRs/MAs included in this overview were reported in their entirety, some reporting deficiencies were found in other sections. In the method section, Item 7 (search strategy) and Item 13 e, f (synthesis methods) have less than 50% response rate. Less than half of the included SRs/MAs were reported on Item 20 d (results of syntheses) in the results section. In addition to this, only 2 (2/11, 18.2%) SRs/MAs provided information on the registration of study protocols, which makes the quality assessment of Item 24 (registration and protocol) reports also unsatisfactory.

### 3.5. Evidence Quality Assessment

The 11 SRs/MAs included in this overview contain 51 outcomes. The results of the quality of evidence assessment showed that 2 items were rated as high quality, 5 items were rated as moderate quality, 24 items were rated as low quality, and the remaining 20 items were rated as extremely low quality. Among the downgrading factors, publication bias (*n* = 46) was the most common downgrading factor, followed by risk of bias (*n* = 30), imprecision (*n* = 26), inconsistency (*n* = 18), and indirectness (*n* = 0). Detailed information on the quality of the evidence was presented in Table 5.

### 3.6. SRs/MAs Outcomes of Intervention

In this overview, we provide a summary and narrative description of the outcome indicators quantitatively assessed by the SRs/MAs. Complete information was presented in Table 6.

### 3.7. Effectiveness Assessment

Seven SRs/MAs [29, 32, 35–39] reported nine outcome indicators on RAS, and 8 of them showed that XFZY improved RAS in patients with CHD, including 2 high-quality pieces of evidence, 1 moderate-quality piece of evidence, 3 low-quality pieces of evidence, and 2 extremely low quality pieces of evidence. Nine SRs/MAs [29, 31–35, 37–39] reported 10 outcome indicators (3 moderate-quality pieces of evidence, 5 low-quality pieces of evidence, and 2 extremely low quality pieces of evidence) on ECG, and only one SR/MA (extremely low quality evidence) showed no efficacy of XFZY compared with CT for improving ECG. Five outcomes (1 moderate-quality piece of evidence and 4 low-quality pieces of evidence) of 4 SRs/MAs [31, 33, 34, 36] reported a significantly higher clinical efficiency rate of XFZY for CHD than the control group. Two SRs/MAs [29, 38] reported that XFZY was effective in reducing LDL-C (1 low-quality piece of evidence and 1 extremely low quality piece of evidence), TC (1 low-quality piece of evidence and 1 extremely low-quality piece of evidence), and one SR/MA [29] reported that XFZY was effective in increasing HDL-C (low-quality evidence). One SR/MA [30] reported that XFZY was effective in treating LVEF (low-quality evidence), LVESD (low-quality evidence), NO (extremely low-quality evidence), ET-1 (extremely low-quality evidence), ICAM-1 (extremely low-quality evidence), SOD (extremely low-quality evidence), MDA (extremely low-quality evidence), BNP (extremely low-quality evidence), and CK-MB (extremely low-quality evidence) in patients with CHD. In addition, the results of one SR/MA [27] showed significant efficacy of XFZY in the treatment of AF (low-quality evidence), DAP (low-quality evidence), WBV (low-quality evidence), PV (extremely low-quality evidence), FB (extremely low-quality evidence), NO (low-quality evidence), and ET-1 (low-quality evidence).

### 3.8. Safety Assessment

One SR/MA [31] quantified the adverse events associated with XFZY for CHD treatment and showed no difference in the incidence of AEs (low-quality evidence) in XFZY compared to controls. In addition, nine SRs/MAs [29, 30, 32–36, 38, 39] gave a narrative description on the low incidence of adverse events in the XFZY group.

## 4. Discussion

TCM has been proven effective in the treatment of CHD, and XFZY is one of the representatives. As the highest level of evidence, SRs/MAs were becoming increasingly important for evidence-based clinical decision-making [40]. Although the number of published SRs/MAs on the XFZY for the treatment of CHD is on the rise, no published overview has thus far put them together and assessed their quality.

### 4.1. Key Findings of This Overview

This overview is the first evaluation of XFZY for CHD-related SRs/MAs using AMSTAR-2, PRISMA 2020, and GRADE. More than half (6/11, 54.5%) of these SRs/MAs were published in the last five years, indicating the growing interest in XFZY for CHD. The included SRs/MAs, on the other hand, were of poor quality.

Based on the details of the AMSTAR-2 assessment, the major factors for the low methodological quality of the included SRs/MAs were as follows: Item 2 (protocol registration, 2/11, 18.2%), Item 7 (exclusion list, 0/11, 0%), and Item 10 (funding sources, 0/11, 0%). Study protocol registration is important when researchers identify topics for SRs/MAs, which helps improve processing transparency and minimize selective reporting bias [41]. A list of excluded literature was not provided for all included SRs/MAs, which may affect the reproducibility of results and undermine the transparency of the study, making it difficult to ensure the reliability of the results. None of the SRs/MAs provided funding resources, which may increase bias in the reporting of clinical trials, as the results of commercially funded studies may be biased toward the institution in question.

For reporting quality, the results of PRISMA 2020 suggest that, as with AMSTAR-2, neither the study protocol nor the source of funding for the RCT was reported in full. In addition, the lack of complete search strategy and sensitivity analysis is also an important reason for the low quality of the report. None of the SRs/MAs provided a complete search strategy for all electronic databases, which renders the studies nonreplicable and may also lead to publication bias. Only 2 (2/11, 18.8%) SRs/MAs had sensitivity analysis, and the absence of sensitivity analysis was detrimental to the stability of the judgmental assessment, which led to a decrease in the credibility of the results.

Regarding evidence quality, only 2 of the 51 outcomes assessed were rated as high quality. Further analysis revealed that publication bias (46/51, 90.2%), risk of bias (30/51, 58.8%), and imprecision (26/51, 51%) were the main factors contributing to the downgrading of the quality of the evidence. The reasons for publication bias may be related to omissions during the literature search and the insufficient number of RCTs on relevant topics. Most of the original RCTs for XFZY treatment of CHD did not explicitly describe the methods of random sequence generation, allocation concealment, and blinding, which may have affected the potency of argumentation of SRs/MAs. The implementation of blinding is difficult due to the particularity of TCM compounds, but scientific methods should be attempted to blind patients, care providers, and outcome assessors. The cause of imprecision is related to the insufficient number of subjects in the RCT, which may be associated with an irrational study design.

Through a narrative overview of the outcome indicators of CHD treated with XFZY, we found that XFZY is effective and safe for CHD, and XFZY is beneficial in relieving angina pectoris, improving electrocardiogram, and reducing blood lipids. In addition, it has potential effects in improving vascular endothelial function and reducing oxidative stress. However, caution is still needed when recommending XFZY for CHD treatment because the low quality of the included SRs/MAs may hinder it from serving as a scientific guidance for clinical practice.

### 4.2. Implications for Future Practice and Research

XFZY exerts its unique advantages in the treatment of CHD through the multitargeted combined action of multiple herbal medicines.

Our findings suggest that XFZY may be a promising complementary therapy for CHD, but due to the overall low quality of the included evidence, the following is strongly advised for future SRs/MAs and RCTs. For TCM-related SRs/MAs, registration on international platforms (e.g., Cochrane Library, PROSPERO, etc.) and/or early publication of protocols is highly recommended. When conducting SRs/MAs, researchers should provide a complete list of search strategies for each electronic database, a list of excluded literature, and the source of funding for the RCT to increase the transparency and reduce the publication bias of the article. To improve the reliability of the results, a sensitivity analysis should be performed. With the development of evidence-based medicine in TCM, it is hoped that researchers will continue to promote the standardization of clinical trial procedures for TCM compounding in the future, including random assignment methods, blinding, and reasonable inclusion of subjects. Clinical researchers should enhance clinical trial top-level design through thorough assessment and sophisticated analysis. The Consolidated Standards of Reporting Trials (CONSORT) should be used to improve the quality of evidence from RCTs [42]. Careful design, rigorous implementation, and complete reporting of RCTs are considered the gold standard for avoiding the risk of bias [43]. In subsequent RCTs of XFZY for CHD, researchers should pay more attention to circulatory-related biochemical indicators or those related to oxidative stress to better investigate the underlying mechanism of XFZY's action. In addition, the dosage and preparation of each Chinese herbal medicine in the XFZY formula should be standardized to make clinical research more scientific.

### 4.3. Strengths and Limitations

This overview is the first to evaluate the current evidence for XFZY in the treatment of CHD from the perspectives of methodological quality, report quality, and evidence quality in all aspects, which can offer helpful advice for clinicians' decision-making as well as suggestions for the upcoming clinical trials. However, this overview also has some limitations, and we found that most of the included SRs/MAs were of poor quality, which may lead to low credibility of the final conclusions. Also, although the assessment has been conducted by two independent assessors, different assessors may have their own judgment on each factor, so the results may vary.

## 5. Conclusions

The evidence suggests that XFZY appears to be an effective and safe method for treating CHD. However, issues with the methodology, quality of the supporting data, and reporting of SRs/MAs and original clinical trials decreased the results' dependability. In order to provide convincing evidence for researchers and clinicians in this field, high-quality clinical studies of XFZY for CHD should be conducted so as to boost the methodological and reporting quality of SRs/MAs.

## Figures and Tables

**Table 1:** Search strategy for the PubMed database.

Query	Search terms
^#^1	“Xuefuzhuyu” OR “xue-fu-zhu-yu” OR “xue fu zhu yu” OR “xuefu zhuyu”

^#^2	“Coronary Disease” [Mesh]

^#^3	“Coronary Diseases” OR “Disease, Coronary” OR “Diseases, Coronary” OR “Coronary Heart Disease”OR“Coronary Heart Diseases” OR “Disease, Coronary Heart” OR “Diseases, Coronary Heart” OR “Heart Disease, Coronary” OR “Heart Diseases, Coronary” OR “Coronary Disease”

^#^4	^#^2 OR ^#^3

^#^5	“Acute Coronary Syndrome” [Mesh]

^#^6	“Acute Coronary Syndromes” OR “Coronary Syndrome, Acute” OR “Coronary Syndromes, Acute” OR “Syndrome, Acute Coronary” OR “Syndromes, Acute Coronary” OR “Acute Coronary Syndrome”

^#^7	^#^5 OR ^#^6

^#^8	“ST Elevation Myocardial Infarction” [Mesh]

^#^9	“ST Segment Elevation Myocardial Infarction” OR “ST Elevated Myocardial Infarction” OR “STEMI” OR “ST Elevation Myocardial Infarction”

^#^10	^#^8 OR ^#^9

^#^11	“Non-ST Elevated Myocardial Infarction” [Mesh]

^#^12	“Non ST Elevated Myocardial Infarction” OR “NSTEMI” OR “Non-ST-Elevation Myocardial Infarction” OR “Infarction, Non-ST-Elevation Myocardial” OR ‘Infarctions, Non-ST-Elevation Myocardial” OR “Myocardial Infarction, Non-ST-Elevation” OR “Myocardial Infarctions, Non-ST-Elevation” OR “Non ST Elevation Myocardial Infarction” OR “Non-ST-Elevation Myocardial Infarctions” OR “Non-ST Elevated Myocardial Infarction”

^#^13	^#^11 OR ^#^12

^#^14	“Angina, Unstable” [Mesh]

^#^15	“Anginas, Unstable” OR “Unstable Anginas” OR “Angina Pectoris, Unstable” OR “Angina Pectori, Unstable” OR “Unstable Angina Pectori” OR “Unstable Angina Pectoris” OR “Unstable Angina” OR “Angina at Rest” OR “Angina, Preinfarction” OR “Anginas, Preinfarction” OR “Preinfarction Angina” OR “Preinfarction Anginas” OR “Myocardial Preinfarction Syndrome” OR “Myocardial Preinfarction Syndromes” OR “Preinfarction Syndrome, Myocardial” OR “Preinfarction Syndromes, Myocardial” OR “Syndrome, Myocardial Preinfarction” OR “Syndromes, Myocardial Preinfarction”

^#^16	^#^14 OR ^#^15

^#^17	“Angina, Stable” [Mesh]

^#^18	“Anginas, Stable” OR “Stable Angina” OR “Stable Anginas” OR “Chronic Stable Angina” OR “Angina, Chronic Stable” OR “Anginas, Chronic Stable” OR “Chronic Stable Anginas” OR “Stable Angina, Chronic” OR “Stable Anginas, Chronic” OR “Angina Pectoris, Stable” OR “Angina Pectori, Stable” OR “Pectori, Stable Angina” OR “Pectoris, Stable Angina” OR “Stable Angina Pectori” OR “Stable Angina Pectoris”

^#^19	^#^17 OR ^#^18

^#^20	^#^4 OR ^#^7 OR ^#^10 OR ^#^13 OR ^#^16 OR ^#^19

^#^21	Meta-Analysis as Topic [Mesh]

^#^22	“Systematic review” OR “meta-analysis” OR “meta analysis” OR “meta-analyses” OR “Review, Systematic” OR “Systematic reviews”

^#^23	^#^21 OR ^#^22

^#^24	^#^1 AND ^#^20 AND ^#^23

**Table 2:** Characteristics of the included SRs/MAs.

Author, year	Trials (subjects)	Intervention Group	Control Group	Quality Assessment	Main Results
Guo-zhong Yi, 2014 [25]	14 (1, 116)	XFZY + CT, XFZY	CT, CT + Placebo	Cochrane Criteria	XFZY combined with CT is more effective than CT alone in improving the clinical symptoms of patients with angina pectoris, especially in patients with stable angina pectoris.
Shiqi Chen, 2022 [26]	16 (1, 171)	XFZY + CT	CT	Cochrane Criteria	In summary, this analysis suggests that XFZY can be used as a representative herbal formula with important clinical applications in improving cardiac and endothelial function as well as LVEF, LVEDD, LVESD, NO, ET-1, and ICAM-1 in acute coronary syndrome.
Shuo Zhang, 2021 [27]	30 (3, 126)	XFZY + CT, XFZY	CT	Cochrane Criteria	XFZY can treat CHD through the combined effect of multiple drugs with significant efficacy and no significant adverse effects. And according to the results, XFZY is more suitable for patients with CHD who have clinical indications such as dyslipidemia, high blood viscosity or vascular endothelial dysfunction.
Xiaochen Yang, 2014 [28]	8 (534)	XFZY + CT, XFZY	CT, CT + Placebo	Cochrane Criteria	Our systematic evaluation suggests that XFZY in combination with CT may have good effects in reducing angina symptoms and improving ECG with few side effects in patients with unstable angina. However, due to the low quality of included trials, no clear conclusions could be drawn. Future more rigorously designed randomized controlled trials with large samples should be conducted.
Zhou Fang, 2016 [29]	22 (1, 951)	XFZY	CT	Cochrane Criteria	Although the present study presents consistent results that XFZY may be more effective than nitrates in the treatment of angina pectoris
Huai Guo, 2017 [30]	12 (1, 252)	XFZY + CT	CT	Cochrane Criteria	In conclusion, XFZY was effective in the adjuvant treatment of unstable angina and significantly improved lipid and inflammatory factor levels in patients.
Jinfeng Liu, 2020 [31]	9 (592)	XFZY + CT	CT	Jadad	The results showed that the efficacy of XFZY plus/minus combined with conventional Western medicine in the treatment of angina pectoris in CHD was better than that of conventional Western medicine, which confirmed the advantages of combined Western and Chinese medicine in the treatment of CHD.
Min Liu, 2016 [32]	8 (598)	XFZY + CT, XFZY	CT	Jadad	XFZY has achieved some efficacy in the treatment of stable angina pectoris.
Yun Liu, 2017 [33]	9 (837)	XFZY + CT	CT	Cochrane Criteria	This systematic review shows that XFZY combined with western medicine is superior to conventional western medicine in the treatment of CHD.
Yao Meng, 2021 [34]	11 (980)	XFZY + CT	CT	Jadad	On the basis of CT, the application of XFZY adjuvant treatment of CHD has a significant effect, which can effectively relieve the symptoms of angina pectoris and improve blood lipid levels.
Guohua Zheng, 2012 [35]	14 (1, 001)	XFZY + CT, XFZY	CT	Jadad	The combination of XFZY and CT can significantly reduce the symptoms of angina pectoris and improve the electrocardiogram in patients with CHD, with few adverse reactions.

**Table 3:** Result of the AMSTAR-2 assessments. Note: y, yes; PY, partially yes; n, no; VL, very low; h, high. Note: key areas are marked in red.

Author, year	Q1	Q2	Q3	Q4	Q5	Q6	Q7	Q8	Q9	Q10	Q11	Q12	Q13	Q14	Q15	Q16	Quality
Guo-zhong Yi, 2014 [25]	Y	PY	Y	PY	N	Y	N	Y	Y	N	Y	Y	Y	Y	Y	N	VL
Shiqi Chen, 2022 [26]	Y	Y	Y	Y	Y	Y	N	Y	Y	N	Y	Y	N	Y	N	Y	VL
Shuo Zhang, 2021 [27]	Y	Y	Y	Y	Y	Y	N	Y	Y	N	Y	Y	Y	N	Y	Y	VL
Xiaochen Yang, 2014 [28]	Y	PY	Y	PY	Y	Y	N	Y	Y	N	Y	Y	Y	Y	N	Y	VL
Zhou Fang, 2016 [29]	Y	PY	Y	PY	Y	Y	N	Y	Y	N	Y	Y	Y	Y	Y	Y	VL
Huai Guo, 2017 [30]	Y	PY	Y	Y	Y	Y	N	N	Y	N	Y	Y	Y	Y	Y	Y	VL
Jinfeng Liu, 2020 [31]	Y	PY	Y	Y	Y	Y	N	Y	Y	N	Y	Y	N	Y	Y	Y	VL
Min Liu, 2016 [32]	Y	PY	Y	Y	N	N	N	Y	Y	N	Y	Y	N	Y	Y	Y	VL
Yun Liu, 2017 [33]	Y	PY	Y	PY	N	N	N	Y	Y	N	Y	N	N	Y	Y	Y	VL
Yao Meng, 2021 [34]	Y	PY	Y	PY	Y	Y	N	Y	Y	N	Y	Y	Y	Y	Y	Y	VL
Guohua Zheng, 2012 [35]	Y	PY	Y	Y	Y	Y	N	Y	Y	N	Y	Y	N	Y	N	N	VL

**Table 4:** Results of the PRISMA checklist. Note: Y, yes; N, no; PY, partially yes.

Section/topic		Items	Guo-zhong Yi, 2014 [25]	Shiqi Chen, 2022 [26]	Shuo Zhang, 2021 [27]	Xiaochen Yang, 2014 [28]	Zhou Fang, 2016 [29]	Huai Guo, 2017 [30]	Jinfeng Liu, 2020 [31]	Min Liu, 2016 [32]	Yun Liu, 2017 [33]	Yao Meng, 2021 [34]	Guohua Zheng, 2012 [35]	Number of yes or partially yes (%)
Title	Title	Item 1	Y	Y	Y	Y	Y	Y	Y	Y	Y	Y	Y	100%
Abstract	Abstract	Item 2	PY	PY	PY	PY	PY	PY	PY	PY	PY	PY	PY	100%
Introduction	Rationale	Item 3	Y	Y	Y	Y	Y	Y	Y	Y	Y	Y	Y	100%
	Objectives	Item 4	Y	Y	Y	Y	Y	Y	Y	Y	Y	Y	Y	100%
Methods	Eligibility criteria	Item 5	Y	Y	Y	Y	Y	Y	Y	Y	Y	Y	Y	100%
	Information sources	Item 6	Y	Y	Y	Y	Y	Y	Y	Y	Y	Y	Y	100%
	Search strategy	Item 7	N	N	N	N	N	N	N	N	N	N	N	0%
	Selection process	Item 8	N	Y	Y	Y	Y	Y	Y	N	N	Y	Y	72.70%
	Data collection process	Item 9	Y	Y	Y	Y	Y	Y	Y	N	N	Y	Y	81.80%
	Data items	Item 10 (a)	Y	Y	Y	Y	Y	Y	Y	Y	Y	Y	Y	100%
		Item 10 (b)	PY	PY	PY	PY	PY	PY	PY	PY	PY	PY	PY	100%
	Study risk of bias assessment	Item 11	Y	Y	Y	Y	Y	Y	Y	Y	Y	Y	Y	100%
	Effect measures	Item 12	Y	Y	Y	Y	Y	Y	Y	Y	Y	Y	Y	100%
	Synthesis methods	Item 13 (a)	Y	Y	Y	Y	Y	Y	Y	Y	Y	Y	Y	100%
		Item 13 (a)	Y	Y	Y	Y	Y	Y	Y	Y	Y	Y	Y	100%
		Item 13 (c)	Y	Y	Y	Y	Y	Y	Y	Y	Y	Y	Y	100%
		Item 13 (d)	Y	Y	Y	Y	Y	Y	Y	Y	Y	Y	Y	100%
		Item 13 (e)	Y	Y	Y	N	N	N	N	N	N	N	N	27.27%
		Item 13 (f)	N	Y	N	N	N	N	N	N	N	N	N	9.09%
	Reporting bias assessment	Item 14	Y	N	Y	N	Y	N	Y	Y	Y	N	N	54.50%
	Certainty assessment	Item 15	N	N	N	N	N	N	N	N	N	N	N	0
Results	Study selection	Item 16 (a)	Y	Y	Y	Y	Y	Y	Y	Y	Y	Y	Y	100
		Item 16 (b)	Y	Y	Y	Y	Y	Y	Y	Y	N	Y	N	81.82%
	Study characteristics	Item 17	Y	Y	Y	Y	Y	N	Y	Y	Y	Y	Y	90.90%
	Risk of bias in studies	Item 18	Y	Y	Y	Y	Y	N	Y	Y	Y	Y	Y	90.90%
	Results of individual studies	Item 19 (a)	Y	Y	Y	Y	Y	N	Y	Y	Y	Y	Y	90.90%
		Item 19 (b)	Y	Y	Y	Y	Y	N	Y	Y	Y	Y	Y	90.90%
	Results of syntheses	Item 20 (a)	Y	Y	Y	Y	Y	N	Y	Y	Y	Y	Y	90.90%
		Item 20 (b)	Y	Y	Y	Y	Y	N	Y	Y	Y	Y	Y	90.90%
		Item 20 (c)	Y	Y	Y	Y	N	Y	Y	Y	Y	Y	Y	90.90%
		Item 20 (d)	N	Y	N	N	N	N	N	Y	N	N	N	18.18%
	Reporting biases	Item 21	Y	N	Y	N	Y	Y	Y	Y	Y	Y	N	72.70%
	Certainty of evidence	Item 22	N	N	N	N	N	N	N	N	N	N	N	0%
Discussion	Discussion	Item 23 (a)	Y	Y	Y	Y	Y	Y	Y	Y	Y	Y	Y	100%
		Item 23 (b)	Y	Y	Y	Y	Y	Y	Y	Y	Y	Y	Y	100%
		Item 23 (c)	Y	Y	Y	Y	Y	Y	Y	Y	Y	Y	Y	100%
		Item 23 (d)	Y	Y	Y	Y	Y	Y	Y	Y	Y	Y	Y	100%
Other information	Registration and protocol	Item 24 (a)	N	Y	Y	N	N	N	N	N	N	N	N	18.18%
		Item 24 (b)	N	Y	Y	N	N	N	N	N	N	N	N	18.18%
		Item 24 (c)	N	N	N	N	N	N	N	N	N	N	N	0%
	Support	Item 25	N	Y	Y	Y	Y	Y	Y	Y	Y	Y	N	81.82%
	Competing interests	Item 26	Y	Y	Y	Y	Y	N	N	N	N	Y	N	54.50%
	Availability of data, code, and other materials	Item 27	Y	Y	Y	Y	Y	Y	Y	Y	Y	Y	Y	100%

**Table 5:** Results of evidence quality. ① The included studies have a large bias in methodology such as randomization, allocation concealment, and blinding. ② The confidence interval overlaps less or the I2 value of the combined results was larger. ③ The sample size from the included studies does not meet the optimal sample size or the 95% confidence interval crosses the invalid line. ④ The funnel chart is asymmetry.

Author, year	Outcomes	Limitations	Inconsistency	Indirectness	Imprecision	Publication bias	Quality
Guo−zhong Yi, 2014 [25]	RAS	0	0	0	0	0	High
	ECG	0	0	0	0	−1④	Moderate
	HDL−C	0	0	0	−1③	−1④	Low
	LDL−C	0	0	0	−1③	−1④	Low
	TG	0	0	0	−1③	−1④	Low
	TC	0	0	0	−1③	−1④	Low

Shiqi Chen, 2022 [26]	LVEF	−1①	0	0	0	−1④	Low
	LVESD	−1①	0	0	0	−1④	Low
	NO	−1①	0	0	−1③	−1④	Very Low
	ET−1	−1①	−1②	0	−1③	−1④	Very Low
	ICAM−1	−1①	−1②	0	−1③	−1④	Very Low
	VCAM−1	−1①	−1②	0	−1③	−1④	Very Low
	CRP	−1①	−1②	0	−1③	−1④	Very Low
	SOD	−1①	0	0	−1③	−1④	Very Low
	MDA	−1①	0	0	−1③	−1④	Very Low
	BNP	−1①	−1②	0	−1③	−1④	Very Low
	CK−MB	−1①	−1②	0	−1③	−1④	Very Low

Shuo Zhang, 2021 [27]	AF	0	−1②	0	0	−1④	Low
	DAP	0	−1②	0	0	−1④	Low
	Clinical Efficiency Rate	0	0	0	0	−1④	Moderate
	ECG	0	0	0	0	−1④	Moderate
	WBV	0	0	0	−1③	−1④	Low
	PV	0	−1②	0	−1③	−1④	Very Low
	FB	0	−1②	0	−1③	−1④	Very Low
	NO	0	0	0	−1③	−1④	Low
	ET−1	0	0	0	−1③	−1④	Low
	AE	0	0	0	−1③	−1④	Low

Xiaochen Yang, 2014 [28]	RAS	−1①	0	0	0	−1④	Low
	ECG	−1①	0	0	−1③	−1④	Very Low
Zhou Fang, 2016 [29]	Clinical Efficiency Rate	−1①	−1②	0	0	0	Low
AE	ECG	−1①	−1②	0	0	0	Low

Huai Guo, 2017 [30]	Clinical Efficiency Rate	−1①	0	0	0	−1④	Low
	CRP	−1①	−1②	0	−1③	−1④	Very Low
	ECG	−1①	0	0	0	−1④	Low

Jinfeng Liu, 2020 [31]	RAS	−1①	0	0	0	0	Moderate
	ECG	−1①	0	0	0	−1④	Low

Min Liu, 2016 [32]	Clinical Efficiency Rate (XFZY + CCT vs CT)	−1①	0	0	0	−1④	Low
	Clinical Efficiency Rate (XFZY vs CT)	−1①	0	0	0	−1④	Low
	RAS (XFZY + CCT vs CT)	−1①	−1②	0	0	−1④	Very Low
	RAS (XFZY vs CT)	−1①	−1②	0	0	−1④	Very Low

Yun Liu, 2017 [33]	RAS	−1①	0	0	0	−1④	Low
	ECG	−1①	0	0	0	−1④	Low

Yao Meng, 2021 [34]	RAS	0	0	0	0	0	High
	ECG	0	0	0	0	−1④	Moderate
	Number of angina attacks	0	−1②	0	−1③	−1④	Very Low
	LDL−C	0	−1②	0	−1③	−1④	Very Low
	TC	0	−1②	0	−1③	−1④	Very Low

Guohua Zheng, 2012 [35]	ECG (XFZY + CT vs CT)	−1①	0	0	0	−1④	Low
	ECG (XFZY vs CT)	−1①	0	0	−1③	−1④	Very Low
	RAS (XFZY + CCT vs CT)	−1①	0	0	0	−1④	Low
	RAS (XFZY vs CT)	−1①	0	0	−1③	−1④	Very Low

**Table 6:** Summary of evidence.

Author, year	Outcomes	Studies (participants)	Heterogeneity	Relative effect (95% CI)	*P*-value	Quality of Evidence
Guo-zhong Yi, 2014 [25]	RAS	12 (992)	0%	RR = 1.29 (1.20, 1.38)	*P* < 0.00001	High
	ECG	9 (683)	0%	RR = 1.37 (1.22, 1.54)	*P* < 0.00001	Moderate
	HDL-C	3 (342)	0%	MD = 0.29 (0.23, 0.35)	*P* < 0.00001	Low
	LDL-C	3 (342)	62%	MD = 1.08 (0.72, 1.44)	*P* < 0.00001	Low
	TG	3 (342)	98%	MD = 0.98 (−0.05, 2.02)	*P*=0.06	Low
	TC	3 (342)	83%	MD = 1.27 (0.63, 1.91)	P=0.0001	Low
Shiqi Chen, 2022 [26]	LVEF	6 (520)	62%	MD = 6.35 (4.20, 8.50)	*P* < 0.00001	Low
	LVESD	5 (416)	98%	MD = −3.48(−5.68, −1.29)	*P*=0.002	Low
	NO	4 (284)	95%	MD = 12.57 (2.95, 22.19)	*P*=0.01	Very Low
	ET-1	5 (344)	99%	MD = −30.93 (−56.59, −5.27)	*P* < 0.00001	Very Low
	ICAM-1	3 (170)	97%	MD = 0.98, (−0.05, 2.02)	*P*=0.02	Very Low
	VCAM-1	3 (170)	98%	MD = −41.07 (−94.39, 12.25)	*P*=0.13	Very Low
	CRP	3 (213)	96%	MD = −1.35 (−3.24, 0.53)	*P*=0.16	Very Low
	SOD	3 (301)	0%	MD = 19.31 (15.96, 22.66)	*P* < 0.00001	Very Low
	MDA	3 (301)	0%	MD = −1.61 (−1.90, −1.33)	*P* < 0.00001	Very Low
	BNP	3 (192)	99%	MD = −49.43 (−71.18, −27.68)	*P* < 0.00001	Very Low
	CK-MB	4 (361)	96%	MD = −10.08 (−14.01, −6.15)	*P* < 0.00001	Very Low
Shuo Zhang, 2021 [27]	AF	9 (1, 349)	98%	MD = −1.01 (−1.31, −0.71)	*P* < 0.00001	Low
	DAP	8 (1, 259)	99%	MD = −1.39 (−1.98, −0.80)	*P* < 0.00001	Low
	Clinical Efficiency Rate	22 (2, 089)	0%	RR = 1.24(1.19, 1.29)	*P* < 0.00001	Moderate
	ECG	7 (619)	18%	RR = 1.31(1.18, 1.46)	*P* < 0.00001	Moderate
	WBV	2 (238)	0%	MD = −0.73 (−0.96, −0.50)	*P* < 0.00001	Low
	PV	3 (343)	93%	MD = −0.46(−0.65, −0.28)	*P* < 0.00001	Very Low
	FB	3 (343)	68%	MD = −0.65 (−0.79, −0.52)	*P* < 0.00001	Very Low
	NO	3 (286)	0%	MD = 4.69 (4.24, 5.13)	*P* < 0.00001	Low
	ET-1	3 (286)	0%	MD = −14.18 (−17.74, −10.61)	*P* < 0.00001	Low
	AE	6 (716)	0%	RR = 0.65(0.38, 1.10)	*P*=0.11	Low
Xiaochen Yang, 2014 [28]	RAS	7 (477)	0%	RR = 1.26 (1.16, 1.38)	*P* < 0.00001	Low
	ECG	4 (276)	0%	RR = 1.20 (1.04, 1.38)	*P*=0.01	Very Low
Zhou Fang, 2016 [29]	Clinical Efficiency Rate	21 (1, 865)	58%	RR = 1.24 (1.16, 1.33)	*P*=0.0004	Low
	ECG	16 (1, 443)	74%	RR = 1.42 (1.22, 1.66)	*P* < 0.00001	Low
Huai Guo, 2017 [30]	Clinical Efficiency Rate	12 (1, 252)	0%	OR = 3.56 (2.49, 5.10)	*P* < 0.00001	Low
	CRP	3 (364)	53%	MD = −0.91 (−1.14, −0.69)	*P* < 0.00001	Very Low
	ECG	8 (758)	15%	OR = 2.76 (1.97, 3.87)	*P* < 0.00001	Low
Jinfeng Liu, 2020 [31]	RAS	9 (754)	0%	RR = 1.24 (1.15, 1.33)	*P* < 0.00001	Moderate
AE	ECG	6 (476)	0%	RR = 1.36 (1.21, 1.53)	*P* < 0.00001	Low
Min Liu, 2016 [32]	Clinical Efficiency Rate (XFZY + CT vs CT)	5 (378)	43%	RR = 1.31 (1.18, 1.44)	*P* < 0.00001	Low
	Clinical Efficiency Rate (XFZY vs CT)	3 (220)	41%	RR = 1.24 (1.09, 1.41)	*P*=0.001	Low
	RAS (XFZY + CT vs CT)	5 (378)	50%	RR = 1.28 (1.06, 1.55)	*P*=0.01	Very Low
	RAS (XFZY vs CT)	2 (120)	55%	RR = 1.41 (1.07, 1.84)	*P*=0.01	Very Low
Yun Liu, 2017 [33]	RAS	9 (837)	0%	OR = 2.83 (2.05, 3.92)	*P* < 0.00001	Low
	ECG	9 (837)	0%	OR = 2.83 (2.56, 5.77)	*P* < 0.00001	Low
Yao Meng, 2021 [34]	RAS	9 (797)	0%	OR = 3.75 (2.42, 5.80)	*P* < 0.00001	High
	ECG	4 (411)	0%	OR = 4.05 (2.21, 7.42)	*P* < 0.00001	Moderate
	Number of angina attacks	3 (390)	97%	SMD = −5.64 (−8.10, −3.18)	*P* < 0.00001	Very Low
	LDL-C	3 (223)	91%	SMD = −1.55 (−2.62, −0.48)	*P*=0.004	Very Low
	TC	3 (223)	59%	SMD = −1.03 (−1.49, −0.56)	*P* < 0.00001	Very Low
Guohua Zheng, 2012 [35]	ECG (XFZY + CT vs CT)	10 (717)	0%	RR = 0.31, (0.21, 0.45)	*P* < 0.00001	Low
	ECG (XFZY vs CT)	2 (171)	0%	RR = 0.93, (0.48, 1.81)	*P* > 0.05	Very Low
	RAS (XFZY + CT vs CT)	8 (632)	0%	RR = 0.61 (0.49, 0.75)	*P* < 0.00001	Low
	RAS (XFZY vs CT)	2 (171)	0%	RR = 0.98 (0.71, 1.36)	*P* > 0.05	Very Low

## Data Availability

The datasets analyzed during the current study are available from the corresponding author upon reasonable request.

## Supplementary Materials

Supplementary Materials Supplementary file 1: definition of Clinical efficiency rate, RAS, and ECG. Supplementary file 2: search strategies for databases other than PubMed.
