# Supplementary material for: The Effect and Safety of Xuefu Zhuoyue Prescription for Coronary Heart Disease: An Overview of Systematic Reviews and Meta-Analyses
Source: Evid Based Complement Alternat Med. 2022 Nov 17;2022:9096940. doi: 10.1155/2022/9096940 (PMC9691319; doi:10.1155/2022/9096940)
Supplement: Supplementary Materials — Supplementary file 1: definition of Clinical efficiency rate, RAS, and ECG. Supplementary file 2: search strategies for databases other than PubMed. [file 9096940.f1.zip › Supplementary file 1 (1).docx]

**Clinical Efficiency Rate:** The evaluation criteria of efficacy refer to the "Guidelines for Clinical Research of New Chinese Medicines", which are divided into markedly effective, effective, ineffective, and aggravated. Total effective rate = (number of markedly effective cases + number of effective cases)/total number of cases × 100%.

**Relief of Anginal Symptoms (RAS):** The improvement of the clinical symptoms of angina pectoris refers to the "Guidelines for Clinical Research of New Chinese Medicines for the Treatment of Chest Arthritis (Angina Pectoris) in Coronary Heart Disease", and is divided into markedly effective, effective, and ineffective according to the number, degree and duration of angina pain attacks, and the total effective number is taken. Marked effect: angina pectoris symptoms disappeared or basically disappeared; effective: the degree of angina pectoris was reduced or the duration was shortened or the number of attacks was reduced; ineffective: the symptoms of angina pectoris did not improve; aggravation: the frequency, degree and duration of pain attacks were aggravated compared with those before treatment. RAS = (number of markedly effective cases + number of effective cases)/total number of cases × 100%.

**Electrocardiogram (ECG):** The improvement of electrocardiogram was divided into markedly effective, effective, and ineffective according to the "Guidelines for Clinical Research of New Chinese Medicines in the Treatment of Thoracic Arthritis (Angina Pectoris) of Coronary Heart Disease", and the total effective number was taken. Markedly effective: ECG returned to normal or almost normal; Effective: ST segment decreased, after treatment, it rose by more than 0.05 mV and did not reach the normal level, and the inverted T wave in the main leads became shallow; or the T wave was flat and became upright; The same as before treatment; aggravation: ST segment decreased by more than 0.05 mV, upright T wave became flat, and flat T wave became inverted. ECG = (number of markedly effective cases + number of effective cases)/total number of cases × 100%.
