# Supplementary material for: The Effect and Safety of Xuefu Zhuoyue Prescription for Coronary Heart Disease: An Overview of Systematic Reviews and Meta-Analyses
Source: Evid Based Complement Alternat Med. 2022 Nov 17;2022:9096940. doi: 10.1155/2022/9096940 (PMC9691319; doi:10.1155/2022/9096940)
Supplement: Supplementary Materials — Supplementary file 1: definition of Clinical efficiency rate, RAS, and ECG. Supplementary file 2: search strategies for databases other than PubMed. [file 9096940.f1.zip › Supplementary file 2 (1).docx]

| **Query** | **Search terms** |
| --- | --- |
| #1 | "Xuefuzhuyu" OR "xue-fu-zhu-yu" OR "xue fu zhu yu" OR "xuefu zhuyu" |
| #2 | [Coronary Disease] explode all trees |
| #3 | "Coronary Diseases" OR "Disease, Coronary" OR "Diseases, Coronary" OR "Coronary Heart Disease" OR "Coronary Heart Diseases" OR "Disease, Coronary Heart" OR "Diseases, Coronary Heart" OR "Heart Disease, Coronary" OR "Heart Diseases, Coronary" OR "Coronary Disease" |
| #4 | #2 OR #3 |
| #5 | [Acute Coronary Syndrome] explode all trees |
| #6 | "Acute Coronary Syndromes" OR "Coronary Syndrome, Acute" OR "Coronary Syndromes, Acute" OR "Syndrome, Acute Coronary" OR "Syndromes, Acute Coronary" OR "Acute Coronary Syndrome" |
| #7 | #5 OR #6 |
| #8 | [ST Elevation Myocardial Infarction] explode all trees |
| #9 | "ST Segment Elevation Myocardial Infarction" OR "ST Elevated Myocardial Infarction" OR "STEMI" OR "ST Elevation Myocardial Infarction" |
| #10 | #8 OR #9 |
| #11 | [Non-ST Elevated Myocardial Infarction] explode all trees |
| #12 | "Non ST Elevated Myocardial Infarction" OR "NSTEMI" OR "Non-ST-Elevation Myocardial Infarction" OR "Infarction, Non-ST-Elevation Myocardial" OR "Infarctions, Non-ST-Elevation Myocardial" OR "Myocardial Infarction, Non-ST-Elevation" OR "Myocardial Infarctions, Non-ST-Elevation" OR "Non ST Elevation Myocardial Infarction" OR "Non-ST-Elevation Myocardial Infarctions" OR "Non-ST Elevated Myocardial Infarction" |
| #13 | #11 OR #12 |
| #14 | [Angina, Unstable] explode all trees |
| #15 | "Anginas, Unstable" OR "Unstable Anginas" OR "Angina Pectoris, Unstable" OR "Angina Pectori, Unstable" OR "Unstable Angina Pectori" OR "Unstable Angina Pectoris" OR "Unstable Angina" OR "Angina at Rest" OR "Angina, Preinfarction" OR "Anginas, Preinfarction" OR "Preinfarction Angina" OR "Preinfarction Anginas" OR "Myocardial Preinfarction Syndrome" OR "Myocardial Preinfarction Syndromes" OR "Preinfarction Syndrome, Myocardial" OR "Preinfarction Syndromes, Myocardial" OR "Syndrome, Myocardial Preinfarction" OR "Syndromes, Myocardial Preinfarction" |
| #16 | #14 OR #15 |
| #17 | [Angina, Stable] explode all trees |
| #18 | "Anginas, Stable" OR "Stable Angina" OR "Stable Anginas" OR "Chronic Stable Angina" OR "Angina, Chronic Stable" OR "Anginas, Chronic Stable" OR "Chronic Stable Anginas" OR "Stable Angina, Chronic" OR "Stable Anginas, Chronic" OR "Angina Pectoris, Stable" OR "Angina Pectori, Stable" OR "Pectori, Stable Angina" OR "Pectoris, Stable Angina" OR "Stable Angina Pectori" OR "Stable Angina Pectoris" |
| #19 | #17 OR #18 |
| #20 | #4 OR #7 OR #10 OR #13 OR #16 OR #19 |
| #21 | "Systematic review" OR "meta-analysis" OR "meta analysis" OR "meta-analyses" OR "Review, Systematic" OR "Systematic reviews" |
| #22 | #1 AND #20 AND #22 |

Search strategy for the Cochrane Library database.

| **Query** | **Search terms** |
| --- | --- |
| #1 | "Xuefuzhuyu" OR "xue-fu-zhu-yu" OR "xue fu zhu yu" OR "xuefu zhuyu" |
| #2 | "Coronary Diseases" OR "Disease, Coronary" OR "Diseases, Coronary" OR "Coronary Heart Disease" OR "Coronary Heart Diseases" OR "Disease, Coronary Heart" OR "Diseases, Coronary Heart" OR "Heart Disease, Coronary" OR "Heart Diseases, Coronary" OR "Coronary Disease" |
| #3 | 'Acute Coronary Syndrome'/exp |
| #4 | "Acute Coronary Syndromes" OR "Coronary Syndrome, Acute" OR "Coronary Syndromes, Acute" OR "Syndrome, Acute Coronary" OR "Syndromes, Acute Coronary" OR "Acute Coronary Syndrome" |
| #5 | #3 OR #4 |
| #6 | 'ST Elevation Myocardial Infarction'/exp |
| #7 | "ST Segment Elevation Myocardial Infarction" OR "ST Elevated Myocardial Infarction" OR "STEMI" OR "ST Elevation Myocardial Infarction" |
| #8 | #6 OR #7 |
| #9 | 'Non-ST Elevated Myocardial Infarction'/exp |
| #10 | "Non ST Elevated Myocardial Infarction" OR "NSTEMI" OR "Non-ST-Elevation Myocardial Infarction" OR "Infarction, Non-ST-Elevation Myocardial" OR "Infarctions, Non-ST-Elevation Myocardial" OR "Myocardial Infarction, Non-ST-Elevation" OR "Myocardial Infarctions, Non-ST-Elevation" OR "Non ST Elevation Myocardial Infarction" OR "Non-ST-Elevation Myocardial Infarctions" OR "Non-ST Elevated Myocardial Infarction" |
| #11 | #9 OR #10 |
| #12 | "Anginas, Unstable" OR "Unstable Anginas" OR "Angina Pectoris, Unstable" OR "Angina Pectori, Unstable" OR "Unstable Angina Pectori" OR "Unstable Angina Pectoris" OR "Unstable Angina" OR "Angina at Rest" OR "Angina, Preinfarction" OR "Anginas, Preinfarction" OR "Preinfarction Angina" OR "Preinfarction Anginas" OR "Myocardial Preinfarction Syndrome" OR "Myocardial Preinfarction Syndromes" OR "Preinfarction Syndrome, Myocardial" OR "Preinfarction Syndromes, Myocardial" OR "Syndrome, Myocardial Preinfarction" OR "Syndromes, Myocardial Preinfarction" |
| #13 | "Anginas, Stable" OR "Stable Angina" OR "Stable Anginas" OR "Chronic Stable Angina" OR "Angina, Chronic Stable" OR "Anginas, Chronic Stable" OR "Chronic Stable Anginas" OR "Stable Angina, Chronic" OR "Stable Anginas, Chronic" OR "Angina Pectoris, Stable" OR "Angina Pectori, Stable" OR "Pectori, Stable Angina" OR "Pectoris, Stable Angina" OR "Stable Angina Pectori" OR "Stable Angina Pectoris" |
| #14 | #2 OR #5 OR #8 OR #11 OR #12 OR #13 |
| #15 | "Systematic review" OR "meta-analysis" OR "meta analysis" OR "meta-analyses" OR "Review, Systematic" OR "Systematic reviews" |
| #16 | #1 AND #14 AND #15 |

1. Search strategy for the Embase database.

SU=(Xuefu Zhuyu Decoction + Xuefu Zhuyu + Xuefu Zhuyu Granules + Xuefu Zhuyu Recipe) AND SU=(Acute Coronary Syndrome + Coronary Heart Disease + Stable Angina + Unstable Angina + Non-ST segment elevation myocardial infarction + ST segment elevation myocardial infarction + chronic coronary disease) AND SU = (systematic review + meta-analysis + meta + meta-analysis)

Search strategy for the CNKI database.

Subject: (Xuefu Zhuyu Decoction or Xuefu Zhuyu or Xuefu Zhuyu Granule or Xuefu Zhuyu Recipe) *Subject: (Acute Coronary Syndrome or Coronary Heart Disease or Stable Angina or Unstable Angina or Non-ST segment elevation myocardial infarction or ST segment elevation myocardial infarction or chronic coronary disease) *topic: (systematic review or meta-analysis or meta or meta-analysis)

Search strategy for the Wanfang database.

(M=Xuefu Zhuyu Decoction+M=Xuefu Zhuyu+M=Xuefu Zhuyu Granules+M=Xuefu Zhuyu Recipe)*(M=Acute Coronary Syndrome+M=Coronary Heart Disease+M=Stable angina+M=unstable angina+M=non-ST-segment elevation myocardial infarction+M=ST-segment elevation myocardial infarction+M=chronic coronary disease)*(M=systematic review+M=meta-analysis+ M=meta+M=meta-analysis)

Search strategy for the Chongqing VIP database.

(Xuefu Zhuyu Decoction OR Xuefu Zhuyu OR Xuefu Zhuyu Granule OR Xuefu Zhuyu Recipe) AND (acute coronary syndrome OR coronary heart disease OR stable angina pectoris OR unstable angina pectoris OR non-ST-segment elevation type Myocardial infarction OR ST-segment elevation myocardial infarction OR chronic coronary disease) AND (systematic review OR meta-analysis OR meta OR meta-analysis)

Search strategy for the Chongqing Chinese Biological Medicine (CBM) database.
